# Supplementary figures and images for: RNase 7 Inhibits Uropathogenic Escherichia coli-Induced Inflammation in Bladder Cells under a High-Glucose Environment by Regulating the JAK/STAT Signaling Pathway
Source: Int J Mol Sci. 2022 May 5;23(9):5156. doi: 10.3390/ijms23095156 (PMC9102358; doi:10.3390/ijms23095156)

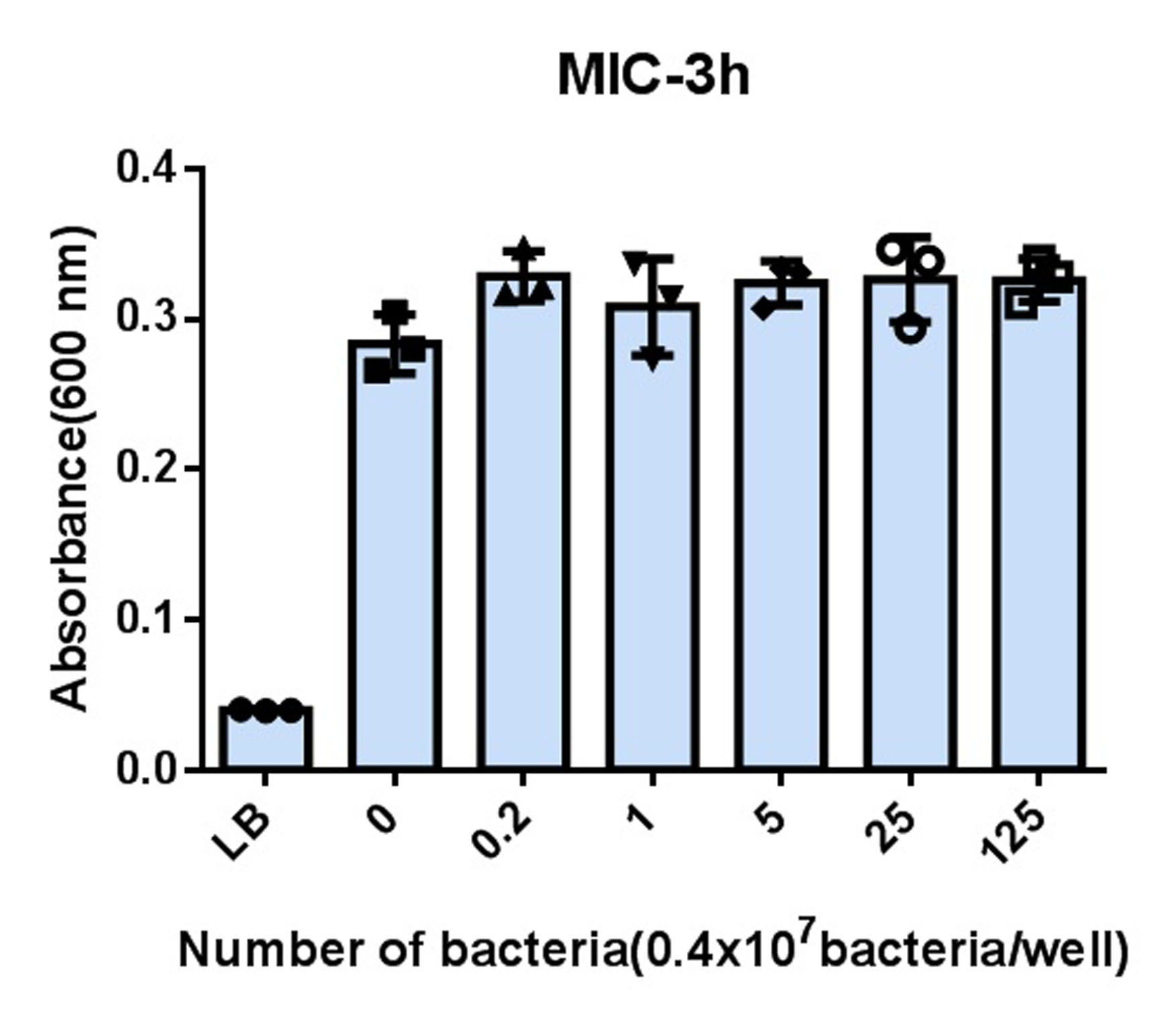

Supplement: Supplementary file 1 [file ijms-23-05156-s001.zip › Supplemental information S1.jpg]

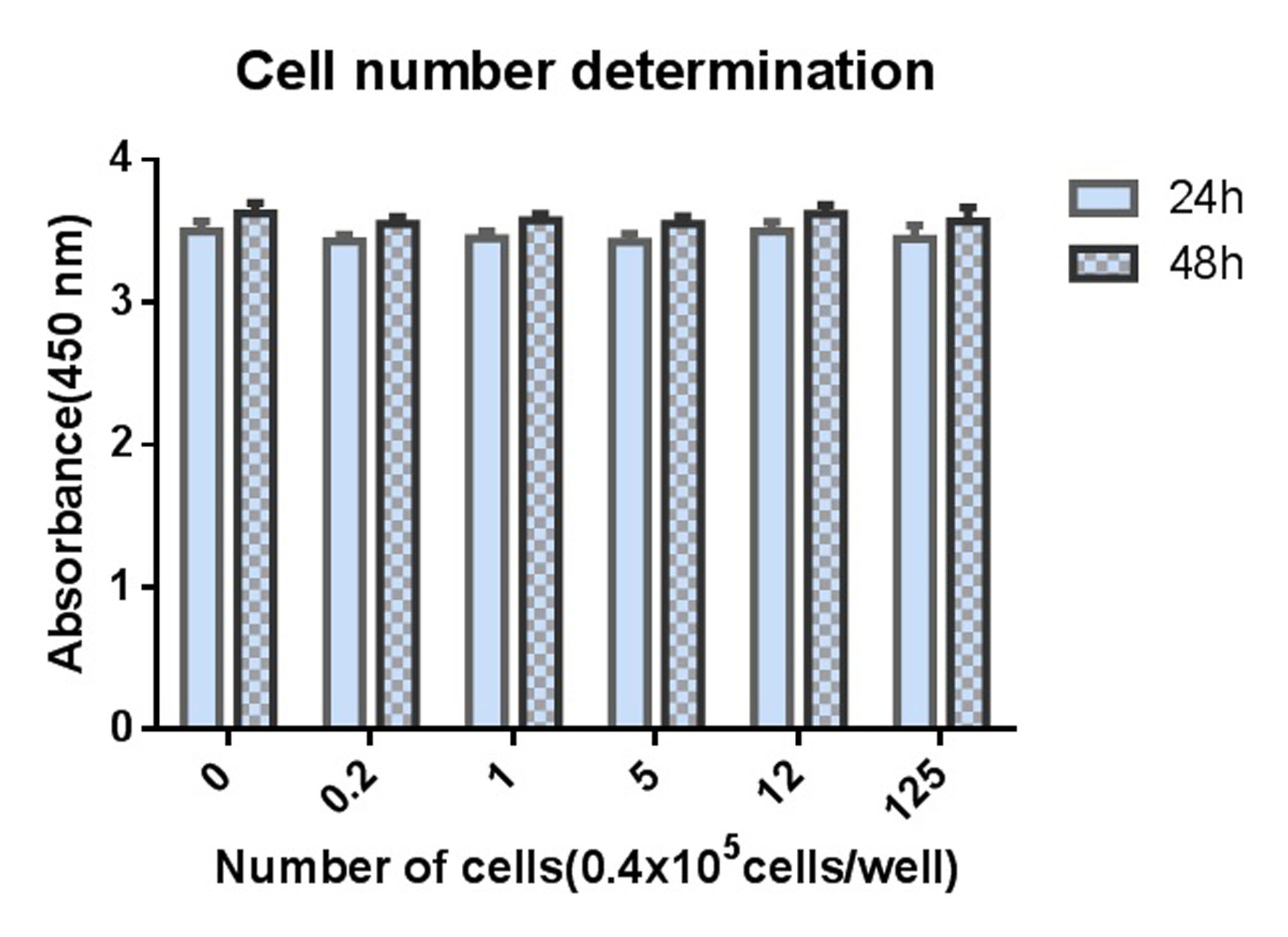

Supplement: Supplementary file 1 [file ijms-23-05156-s001.zip › Supplemental information S2.jpg]
